# Large scale genomic analysis shows no evidence for pathogen adaptation between the blood and cerebrospinal fluid niches during bacterial meningitis

**DOI:** 10.1099/mgen.0.000103

**Published:** 2017-01-31

**Authors:** John A. Lees, Philip H. C. Kremer, Ana S. Manso, Nicholas J. Croucher, Bart Ferwerda, Mercedes Valls Serón, Marco R. Oggioni, Julian Parkhill, Matthijs C. Brouwer, Arie van der Ende, Diederik van de Beek, Stephen D. Bentley

**Affiliations:** ^1^​ Pathogen Genomics, Wellcome Trust Sanger Institute, Hinxton, UK; ^2^​ Department of Neurology, Center for Infection and Immunity Amsterdam (CINIMA), Academic Medical Center, Amsterdam, The Netherlands; ^3^​ Department of Genetics, University of Leicester, Leicester, UK; ^4^​ Department of Infectious Disease Epidemiology, Imperial College London, London, UK; ^5^​ Department of Medical Microbiology, Center for Infection and Immunity Amsterdam (CINIMA), Academic Medical Center, Amsterdam, The Netherlands; ^6^​ Netherlands Reference Laboratory for Bacterial Meningitis, Academic Medical Center, Amsterdam, The Netherlands

**Keywords:** meningitis, within-host evolution, *Streptococcus pneumoniae*, *Neisseria meningitidis*, pan-genome calling, ivr locus

## Abstract

Recent studies have provided evidence for rapid pathogen genome diversification, some of which could potentially affect the course of disease. We have previously described such variation seen between isolates infecting the blood and cerebrospinal fluid (CSF) of a single patient during a case of bacterial meningitis. Here, we performed whole-genome sequencing of paired isolates from the blood and CSF of 869 meningitis patients to determine whether such variation frequently occurs between these two niches in cases of bacterial meningitis. Using a combination of reference-free variant calling approaches, we show that no genetic adaptation occurs in either invaded niche during bacterial meningitis for two major pathogen species, *Streptococcus pneumoniae* and *Neisseria meningitidis*. This study therefore shows that the bacteria capable of causing meningitis are already able to do this upon entering the blood, and no further sequence change is necessary to cross the blood–brain barrier. Our findings place the focus back on bacterial evolution between nasopharyngeal carriage and invasion, or diversity of the host, as likely mechanisms for determining invasiveness.

## Abbreviations

CNV, copy-number variation; CSF, cerebrospinal fluid; HPD, highest posterior density; MLST, multilocus sequence type; NRLBM, Netherlands Reference Laboratory for Bacterial Meningitis; SNP, single-nucleotide polymorphism.

## Data Summary

1. Read data, assembled and annotated contigs are deposited in the European Nucleotide Archive (ENA): study accession number ERP004245 (url – www.ebi.ac.uk/ena/data/view/ERP004245).
2. Code and R scripts used to carry out this analysis can be accessed at https://github.com/johnlees/paired-samples.
3. Sample metadata used has been deposited in Figshare: DOI: 10.6084/m9.figshare.4329809 (url – http://dx.doi.org/10.6084/m9.figshare.4329809).

## Impact Statement

We have analysed the genomes from bacterial pathogen isolates from cases of meningitis in 869 Dutch adults, focusing on comparing pairs of isolates from the patient’s blood and their cerebrospinal fluid. Previous research has been on only a single patient, but showed possible signs of adaptation to treatment within the host over the course of a single case of disease. By sequencing many more such paired samples, and including two different bacterial species, we were able to determine that adaptation of the pathogen does not occur after bloodstream invasion during bacterial meningitis. Overall, our findings indicate that evolution after invasion in bacterial meningitis is not a major contribution to disease pathogenesis. Future studies should involve more extensive sampling between the carriage and disease niches, or the variation of the host.

## Introduction

Bacterial meningitis is a severe inflammation of the meninges surrounding the brain as a response to the presence of bacteria [1]. This inflammation can compromise brain function, requiring immediate admission to hospital. In European countries, the bacteria that most frequently cause meningitis are *Streptococcus pneumoniae* and *Neisseria meningitidis* [2].

The route of infection varies depending on the species of bacteria, although in the majority of invasive cases the final stage is from blood to cerebrospinal fluid (CSF) [1]. These respiratory pathogens are carried asymptomatically in the nasopharynx by a proportion of the population at any given time [3, 4]. In a small number of cases, commensal nasopharyngeal bacteria may invade the blood through a single cell bottleneck (bacteraemia) [5], then cross the blood–brain barrier into the CSF where they cause meningitis [6]. In some meningitis patients, the CSF may be invaded directly due to CSF leakage or otitis media [7], in which case the progression of bacteria after carriage is reversed: CSF to blood.

Previously, it was thought that mutation rates in bacterial genomes were low, and as such there would be no change within a single host [8]. Through whole-genome sequencing, however, variation over the course of a single bacterial infection was found to exist [9, 10]. Additionally, many studies sequencing bacterial populations of various different species gave estimates of mutation rates three orders of magnitude higher than previously expected [11–13]. These new estimates of mutation rate were also supported by evidence that DNA sequence variation can occur over the course of a single infection [14].

Such within-host variation has been shown to occur through a variety of mechanisms, such as recombination [15], gene loss [16, 17] and variation in regulatory regions [18–20]. The rapid variation that occurs in these regions of the genome can increase the population’s fitness as the bacteria adapt to the host environment [21, 22], and potentially affect the course of disease [23]. Previous studies have shown variation between strains even during the rapid clinical progression of bacterial meningitis [24, 25].

It is possible that bacteria inhabiting the nasopharynx are already well adapted for CSF invasion. However, genetic variants that enable invasion of the CSF are not expected to be under positive selection, since invasion is an evolutionary dead end for the bacterium. Studies of carriage alone will therefore be unable to detect selection during invasion. Current knowledge on within-host variation during invasive disease is mostly focused at the serotype and multilocus sequence type (MLST) level, and lacks the resolution and sample size to be able to address this question [26–28]. Though the only whole-genome-based study suggests there is no difference between blood and CSF populations (at the gene level) in *S. pneumoniae* [29], we believe larger sample sizes are needed to better answer this question.

We hypothesize that bacterial variation may also occur during the invasive phase of meningitis. We have previously reported in a single patient that the bacteria appeared to adapt to the distinct conditions of blood and CSF [24]. These are very different niches from that of nasopharyngeal carriage where this variation is well documented [30], not least because the bacteria are exposed to different immune pressures [31] and have less time over which to accumulate mutations.

To look for adaptation to these niches, we used samples from the MeninGene study [32, 33], based at the Academic Medical Center Amsterdam (The Netherlands). A total of 938 patients recruited to the study had culture-positive bacterial meningitis with samples collected from both their blood and CSF (breakdown by species is shown in Table S1, available in the online Supplementary Material). By whole-genome sequence analysis of large numbers of paired bacterial isolates cultured from these samples, we have been able to test for convergent adaptation occurring during the course of the disease.

## Methods

### Strain collection and culture

All isolates were retrieved from the collection of the Netherlands Reference Laboratory for Bacterial Meningitis (NRLBM). The NRLBM receives nationwide isolates from patients with bacterial meningitis as part of the laboratory-based surveillance of bacterial meningitis. Isolates were originally cultured from patient’s blood and/or CSF according to the standard practice of diagnosis of bacterial meningitis by the submitting medical microbiology laboratories associated with hospitals. Isolates were cultured on lysed blood agar (chocolate agar) in Bijou bottles, provided by the NRLBM and subsequently submitted to that laboratory.

After receipt by the NRLBM, *N. meningitidis* isolates were recultured through either one or two passages on chocolate agar plates and *S. pneumoniae* isolates were recultured on blood agar (5 % sheep blood in agar) plates. All plates were incubated overnight at 37 °C in a humidified atmosphere of 5 % CO_2_. Subsequently, cultures were collected and suspended in glycerol (8 % v/v) peptone and stored at −70 °C. Isolates were recultured from frozen stock on blood agar plates, from which DNA was extracted directly. Between each culturing step, a plate sweep was taken, rather than single-colony picks.

### Reference-free variant calling

We extracted DNA from positive blood and CSF cultures from adults with bacterial meningitis in the Netherlands from 2006 to 2012, and sequenced this with 100 bp paired-end reads using the Illumina HiSeq platform. To avoid reference bias, and missing variants in regions not present in an arbitrarily chosen reference genome, we then performed reference-free variant calling between all sequence pairs of isolates using two methods: the ‘hybrid’ method [34] and cortex method [35]. The former uses *de novo* assembly of the CSF sequence reads, mapping reads from both the blood and CSF samples back to this sequence, then calling variants based on this mapping. cortex uses an assembly method that keeps track of variation between samples as it traverses the de Bruijn graph.

In the hybrid method, we created draft assemblies of the CSF samples using spades v3.5 [36], corrected these with sspace and gapfiller [37], then mapped reads from both blood and CSF samples to this reference using snap [38], followed by variant calling with bcftools v1.1 [39] using the command samtools mpileup -C 50 m 2 F 0.0005 -d 1000 t DP, SP -g -p -L 1000 f assembly.fa mapping.bam | bcftools call -vm -P 1e−3 samples.txt. Variants with QUAL <50, MQ <30, SP >30, MSQB <0.001, RPB <0.001 or DP <4 were filtered out.

For cortex, we first error corrected sample reads using quake [40], preventing false-positive calls supported by very low coverage of reads. The joint workflow of cortex was then used with each set of corrected reads in its own path in the de Bruijn graph, and bubble calling was used to produce a second set of variants between samples. Single-nucleotide polymorphisms (SNPs) in the error-corrected reads were also called using the graph-diff mode of sga [41].

### Simulations of closely related genomes

As the rate of variation is very low, we needed to ensure we had sufficient power to call variants and did not suffer from an elevated false-negative rate. We did this by simulating evolution of *S. pneumoniae* genomes along the branch of the tree between *S. pneumoniae* R6 [42] and the common ancestor with *Streptococcus mitis* B6 [43] (Fig. S1). The rates in the generalized time-reversible (GTR) matrix and insertion/deletion frequency distributions were estimated by aligning the R6 and B6 reference sequences with progressive cactus [44]. A mean of 200 mutations with these rates was created in 100 sequences, and Illumina paired-end read data at 200× coverage simulated using pirs [45]. Variants between these sequences and a draft R6 assembly from simulated read data were then called using both of the above methods; comparison with the mutations known to be introduced allowed power and false-positive rate to be calculated – separately for SNPs (single base substitution) and INDELs (one or more bases inserted or deleted).

In addition to *in silico* simulation, we cultured blood/CSF paired strains 4038 and 4039 [24], and resequenced them using the same 100 bp Illumina paired-end sequencing as the rest of the isolates in the study. The genomes of strains 4038 and 4039 have been exhaustively analysed using multiple sequencing technologies (Illumina, 454 and capillary sequencing), so represent high-quality positive-control data to assess the calling methods. Both methods were tested on these data.

The highest power was achieved using hybrid mapping for SNPs and Cortex for INDELs: median power for calling SNPs was 90 % using hybrid mapping, and 74 % for INDELs using cortex (Figs S2 and S3). This combination of methods, mapping for SNPs and cortex for INDELs, was therefore used across all samples. When applied to the paired strains 4038/4039, the same mutations as originally reported were recovered, plus a 37 bp insertion in *cysB* that was found to have been introduced during culturing.

We used simulations to compare against a simple method of mapping against an arbitrary reference, in this case TIGR4 [46]. We found our reference-free method had greater power, especially for INDELs (Fig. S4), and a markedly reduced false-positive rate. We also tested an assembly method alone to compare gene presence and absence, but this too suffered from a vastly elevated false-positive rate (Fig. S5).

### Tests for genes, intergenic regions and genotypes enriched for mutation

To scan for repeated variation, the number of mutations in each coding DNA sequence (CDS) annotation (adjusted for CDS length) was counted. We then performed a single-tailed Poisson test using the genome-wide mutation rate per bp multiplied by the gene length as the expected number of mutations. The resulting *P* values we corrected for multiple testing using a Bonferroni correction with the total number of genes tested as the *m* tests. We reported those CDS with an adjusted *P* value less than the significance level of 0.05. For intergenic regions, any region with more than one variant is reported.

To test whether certain genotypic backgrounds are associated with a higher number of mutations that occurs post-invasion, a linear fit of each MLST against number of mutations between blood and CSF isolates was performed. The *P* values of the slope for each MLST were Bonferroni corrected; at a significance level of 0.05 no MLST was associated with an increased number of mutations. For genes, the same test was performed, except samples were coded as one and zero based on whether they had a mutation in the gene being tested or not. We performed a logistic regression for each gene with over ten mutations reaching significance in the Poisson test: no genes being mutated post-invasion were associated with an MLST.

### Copy-number variation (CNV)

We called CNVs between samples by first mapping each species to a single reference genome (ATCC 700669), then fitting the coverage of mapped reads with a mixture of Poisson distributions [47]. Regions of 1 kb were ranked by the number of sample pairs containing a discordant CNV call, as defined by the integer copy number being different between blood and CSF samples. We then inspected the top 5 % of these regions.

In *S. pneumoniae*, the most frequently varying region was due to poor quality mapping of a prophage region. The only other region with *P*<0.05 was a change in copy number of 23S rRNA seen in a small number of sample pairs. In *N. meningitidis*, mismapping in the *pilE*/*pilS* region accounted for the only CNV change.

### Variant direction and effect annotation

To then be able to compare between samples using a consistent annotation, we mapped the called variants to the ATCC 700669 reference [48] for *S. pneumoniae*, and MC58 reference [49] for *N. meningitidis*. This was done by taking a 300 base window around each variant and using blastn on these with the reference sequence. ‘Directionality’ was then relative to the reference used. We used vep [50] to annotate consequences of each variant. The variants were visualized by plotting variant start positions between all pairs against a reference genome, which allowed identification of clustering of variation in unannotated regions.

### *ivr* locus allele determination directly measured from clinical samples

As the rate of variation at this locus is fast compared to other types of variation measured, we checked that culturing the bacteria did not cause the allele to change from what was observed in the original clinical sample. We therefore extracted DNA from a subset of 53 of 674 paired clinical CSF samples and the respective bacterial isolates.

Allele prevalence was quantified using a combined nested PCR protocol based on PCR amplification of the *ivr* locus, using primers 5′-CCATTATCTATAGGCGTATTTTTACG-3′- and 5′-FAM-GGAAACTGAGATATTTCGTGGTG-3′ (where FAM is 6-fluorescein amidite), with digestion with DraI and PleI, followed by quantitative analysis of banding patterns on a capillary electrophoresis system [19]. Allele prevalence was identical between the original clinical sample and cultured bacteria in 50 out of the 53 samples. The predictive power of the *in vitro* detected *ivr* allele prevalence in a pneumococcal culture for the original allele prevalence within the clinical sample is therefore sufficient to draw conclusions from.

### *ivr* locus allele determination from genomic data

There are six possible alleles A–F at this locus, though due to the high variation rate and structural rearrangement mediating the change the allele cannot reliably be determined using assembly and/or standard mapping of short read data. Instead, mates of reads mapping to the reverse strand of the conserved 5′ region were extracted for each sample, and mapped with blat [51] to the possible alleles in position 1. This forms a vector *r_i_* of length two for each sample *i*, with the number of reads mapped to 1.1 and 1.2. Similarly, to determine the 3′ allele (position 2), pairs of reads mapping to each of the reverse strand of allele 1.1 and the forward strand of allele 1.2 were extracted and mapped to the three possible alleles in position 2 (Fig. S6). This forms a vector *q_i_* of length six for each sample *i*, with the number of reads mapped to each allele A–F.

From mapping, we found 621 sample pairs had at least one read mapping to an allele of the *ivr* locus *hsdS* gene (Table S2). Those without any reads mapping had either a deletion of one component of the locus, or a large insertion mediated by the *ivr* recombinase.

### Bayesian model for *ivr* allele

We first modelled the state of the 5′ allele (TRD1.*j*) only. For the two possible alleles 1.1 and 1.2, the number of reads mapping to each allele (a 2-vector *r_i_*) was used as the number of successes in multinomial distribution *z_c_*(*c* – index for niche). From these, we inferred the proportion of each allele in each individual sample π_i_, and in each niche overall μ_c_. This was done by defining Dirichlet priors expressing the expected proportion of an allele in a given sample π_i_ to be drawn from a Dirichlet hyperprior representing the proportion of the allele that is found in each niche as a whole μ_c_. The κ parameter sets the variance of all the individual sample allele distributions π_ic_ about the tissue mean μ_c_, with a higher κ corresponding to a smaller variance. This model is represented in Fig. S7. The hyperparameter *A*μ, which encodes the total proportion of each allele we expected to see over all samples, was set to the mean amount of the allele observed from the long-range PCR in a subset of 53 paired samples, as described above.

The observed number of reads mapping to each allele, prior distributions defined above, and structure of the model in Fig. S7 defines a likelihood that can be used to infer the most likely values of the parameters of interest π and μ. We used Rjags to perform Markov-Chain Monte Carlo (MCMC) sampling to simulate the posterior distribution of these parameters. We used three different starting points, and took and discarded 30 000 burn-in steps, followed by 45 000 sampling steps. Noticeable auto-correlation was seen between consecutive samples, so only every third step in the chain was kept in sampling from the posterior. We manually inspected plots of each hyperparameter value and mean at each point in the chain, as well as the Gelman and Rubin convergence diagnostic, which showed that the chain had converged over the sampling interval (Fig. S8).

To model both the 5′ end (TRD 1.1 and 1.2) and the 3′ end (TRD 2.1, 2.2 and 2.3) together, so each isolate *i* is represented by an allele A–F, for each isolate the total number of reads mapping *n_i_* was drawn from the distribution in equation 1:

(1) $$n_{i}∼\pi_{i}\cdot r_{i}$$

where *j* is the index of the TRD region, *r_ij_* is the number of reads in sample *i* that had a mate pair downstream from TRD1.*j* mapping to any TRD2 region, and π*_i_* is the posterior for allele frequency in the sample.

The distribution for the number of reads mapping to each allele was defined as in equation 2:

(2) $$z_{i,j}∼\left\{ \begin{array}{cc} n_{i}\cdot \frac{q_{i,j}}{\vec{q_{i}}}\cdot \pi_{i,1.1}, & ifj\in A,B,E\\ n_{i}\cdot \frac{q_{i,j}}{\vec{q_{i}}}\cdot \pi_{i,1.2}, & ifj\in C,D,F \end{array}\right.$$

where *q_i_* is a vector of length six that contains the number of reads mapped to each allele A–F as described above, and π, *i* and *n* are as previously. A single sample for *z* was taken for each isolate *i*. This 6-vector *z_ij_* is then used as the observed data in the same model as above to infer π_i_, and μ_c_ for the whole locus allele (A–F) rather than just the 5′ end.

For the 5′ allele (TRD1.*j*), a model using a single κ parameter rather than a κ indexed by tissue *c* was preferred (change in deviance information criterion [52] ΔDIC = −0.523). For the 3′ allele (TRD2.*j*), a model with a single κ parameter did not converge. A model with κ indexed by allele was used instead.

### Diversity of *ivr* allele within samples

As the speed of inversion is rapid, the subsequent polymorphism of this locus was also used to evaluate our assumptions about diversity of the bacterial population within each niche. We calculated the Shannon index of each sample’s vectors π_CSF_ and π_blood_ to measure diversity of the sample in each niche. The mean Shannon index across CSF samples was 1.01 [95 % highest posterior density (HPD) 0.39–1.51] and 0.98 (95 % HPD 0.35–1.55) in the blood. Looking at each sample pair individually, the difference between diversity in each niche appears normally distributed with a mean of zero (Fig. S9). Together, these observations suggest a similar rate of diversity generation in each niche. This is in line with our assumption that the two populations have similar mutation rates, and a similar number of generations between being founded and being sampled.

## Results

We made assumptions about the evolution of bacteria within the host, under which we discuss the power of pairwise comparisons between single colonies taken from each niche to capture repeated evolution occurring post-invasion. (i) There is a bottleneck of a single bacterium upon invasion into the first sterile niche (usually blood), which then founds the post-invasion population [5, 53]. (ii) A large invasive population is quickly established, as the population size approaches the carrying capacity of the blood/CSF. The population size is large enough for selection to operate efficiently. (iii) As infection occurs in a mass transport system, populations are well mixed without any substructure. Therefore, the effective population size equals the census population size. (iv) The bacterial growth rate within blood and CSF is similar.

Initially the population size is small, so selection is inefficient and the population-wide mutation rate is low. However, the eventual carrying capacity (the maximum number of cells) of the blood and CSF are large enough (>1.5×10^5^ c.f.u.) [54, 55] for beneficial mutations to fix rapidly. Due to the short generation time of around an hour [56], this carrying capacity is reached early in the course of the disease (after 1–2 days) [57].

Crucially, population sizes where selection acts efficiently [58] are reached even earlier than this – a few hours after invasion. Therefore, mutations with a selective advantage occurring after the first stages of infection will eventually become fixed in the niche’s population. So, sequence comparison between colony picks from each niche is likely to find adaptation that has occurred post-invasion.

Similarity of the bacterial growth rate within blood and CSF is an important assumption because in 45 % of the pneumococcal cases there was evidence that CSF invasion happened before blood invasion (patients had a documented prior CSF leak, otitis media or sinusitis) [59, 60]. This allowed us to search for post-adaptation invasion that happens in either direction in this species. We investigated the validity of this assumption using analysis of data on the *ivr* locus (see Methods).

Finally, we considered whether the culturing process would bias our results. In *S. pneumoniae*, we found that two additional passages of the previous sample pair resulted in one additional insertion. In *N. meningitidis*, a low rate of variation, no selection on phase-variable regions and no variation of other regions have been observed during culture steps [61–63]. We therefore concluded that there would be minimal bias introduced during culturing, and that which was introduced would increase the frequency of mutations between pairs without bias towards either blood or CSF.

### No repeated post-invasion adaptation in coding regions across species

We performed comparisons between the pan-genome of each pair of blood and CSF isolates, using reference-free variant calling techniques (see Methods). The method was evaluated using simulated data, giving us confidence that it could detect the small amounts of variation expected between each isolate pair (Figs S2 and S3).

**Fig. 1.**
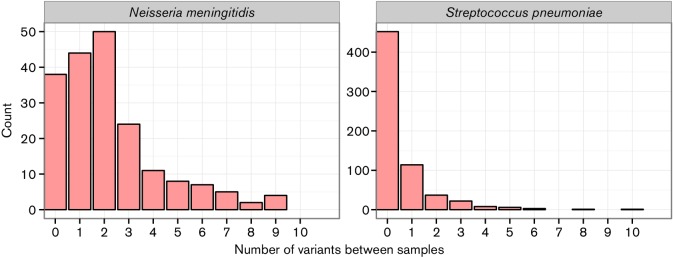
Histograms binned by number of variants between a blood/CSF sample pair, for both pathogens. Total pairs analysed: 674 *S. pneumoniae*, 195 *N. meningitidis*. SNPs are from mapping, INDELs are from Cortex. Three *S. pneumoniae* and one *N. meningitidis* sample with over 10 variants are not shown.

For each species, we then counted the number of variants of any type between each blood/CSF isolate pair taken from a patient (Fig. 1). In *S. pneumoniae*, 452 of 674 paired samples (67 %) were identical. The distribution of number of variants between isolate pairs was roughly Poisson (mean=0.547), excluding outliers. Variation between *N. meningitidis* pairs also followed a roughly Poisson distribution (mean=2.34), which when compared to *S. pneumoniae* showed a higher number of variants between blood and CSF isolates (Wilcoxon rank-sum test, W=25790, *P* value <10^−10^) such that most pairs had at least one variant between the blood and CSF samples.

In both species, the mutations that do exist, if they cause the same functional change, could represent a signal of adaptation. To determine whether this was the case, we counted the number of times each gene contained variation between the blood and CSF isolate over all the pairs collected, and used a Poisson test to determine whether this was more than expected for each gene (see Methods).

For *S. pneumoniae*, the results are shown in Table 1. The *dlt* operon, responsible for d-alanylation in teichoic acids in the cell wall [31, 64, 65], was the most frequently mutated region: 36 mutations in 31 sample pairs (Poisson test *P*<10^−10^). This occurred in only 5 % of samples, so adaptation to a niche due to variation in genes is not common.

**Table 1.** Genes containing significantly repeated mutations between blood and CSF isolate pairs in *S. pneumoniae* Ordered by increasing *P* value; locus tags refer to the D39 genome, if present.

Gene name	Gene length (bp)	Mutations between blood and CSF	*P* value
*pde1* (SPD_2032)	1973	19	<10^−10^
*dltD* (SPD_2002)	1269	13	<10^−10^
*dltB* (SPD_2004)	1245	12	<10^−10^
*dltA* (SPD_2005)	1551	11	<10^−10^
*clpX* (SPD_1399)	1233	7	1.3×10^−8^
*wcaJ* (SPD_1620)	693	6	3.4×10^−8^
*cysB* (SPD_0513)	909	5	1.6×10^−5^
*cbpJ*	1122	5	4.7×10^−5^
*amiC* (SPD_1670)	1332	4	6.0×10^−3^
*marR*	435	3	9.6×10^−3^
*fhuC*	519	3	1.6×10^−2^

**Fig. 2.**
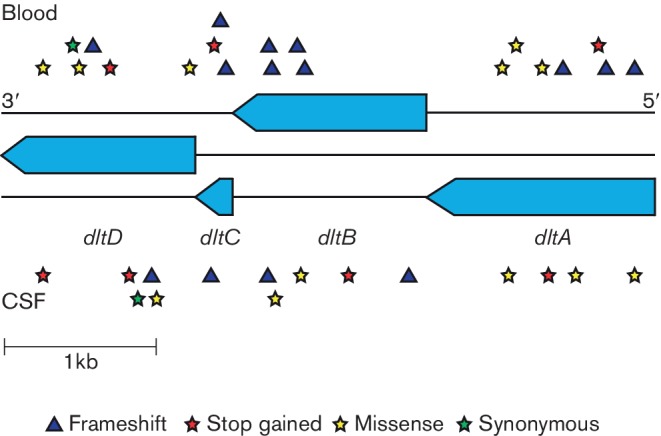
Mutations observed between all paired samples in the *dlt* operon. The operon consists of four genes in the three reading frames of the reverse strand. Mutations, displayed by type, in the blood strains are shown above the operon, and in the CSF strains below the operon.

To investigate whether this represented adaptation to either blood or CSF, we annotated the effect of these variants, and determined whether they were specific to a niche. We mapped them to the R6 *S. pneumoniae* strain, which has a functional *dlt* operon and was therefore assumed to be the ancestral state. There was no directionality to the mutations: 19 occurred in the blood, and 11 in the CSF (*P*=0.2). Only seven of the patients infected by these strains showed signs of blood invasion before CSF invasion (sinusitis or otitis); this also did not show directionality. The nature of the mutations is shown in Fig. 2 and Table S3. Most of these mutations would be expected to cause a loss of function in the operon. Though this suggests this locus has a deleterious effect in invasive disease generally, the lack of directionality to the mutations means it does not show evidence of adaptation to either the blood or CSF specifically.

In all the other genes in Table 1 the variants are non-synonymous SNPs distributed evenly between blood and CSF; therefore, also showing no adaptation specific to either niche.

The most frequently mutated genes between pairs in *N. meningitidis* are shown in Table 2. Top ranked are those relating to the pilus: *pilE* (19), *pilC* (6) and *pilQ* (4). Pilus genes are associated with immune interaction [66], and are therefore expected to be under diversifying selection; an excess of non-synonymous mutations (dN/dS=1.39; *P*=0.024) is consistent with this. The other notable gene with more mutations than expected in *N. meningitidis* was *porA*, encoding a variable protein that is a major determinant of immune reaction [67], in which 12 samples had frameshift mutations in one of two positions. Phase variation in the gene’s promoter region, affecting its expression, is discussed in more detail below.

**Table 2.** Genes containing significantly repeated mutations between blood and CSF isolate pairs in *N. meningitidis* Ordered by increasing *P* value; locus tags refer to the MC58 genome, if present.

Gene name	Gene length (bp)	Mutations between blood and CSF	*P* value
*pilE* (NMB0018)	384	18	<10^−10^
*lgtC*	189	16	<10^−10^
*hyaD*	327	14	<10^−10^
*oatA*	1869	19	<10^−10^
*hpuB* (NMB1668)	2382	17	<10^−10^
*porA* (NMB1429)	1178	12	<10^−10^
*lgtA* (NMB1929)	1050	10	<10^−10^
*kfoC*	360	7	<10^−10^
*cotSA*	1134	7	9.2×10^−9^
*ssa1*	3252	6	3.9×10^−4^

The mutations in Table 2 show no association with blood or CSF specifically, so do not represent adaptation to either niche. Genetic variation in *pilE*, *hpuA*, *wbpC*, *porA* and *lgtB* within-host has been observed previously in a single patient with a hypermutating *N. meningitidis* infection [25]. These coding sequences overlap with those in Table 2, which also suggests an elevated background mutation rate in these sequences, rather than strong selection between the blood and CSF niches.

### No evidence for repeated adaptation in intergenic regions in *S. pneumoniae* and *N. meningitidis*

Our previous result suggesting adaptation from blood to CSF was an intergenic change affecting the transcription the *patAB* genes, encoding an efflux pump [24]. In general, it is known that in pathogenic bacteria a common form of adaptation is mutation in intergenic regions, which may affect global transcription levels, causing a virulent phenotype [68, 69], antimicrobial resistance [70] and changing interaction with the host immune system [71]. Changes in these regions have previously been shown to display signs of adaptation during single cases of bacterial disease [20].

We therefore separately investigated the mutations in non-coding regions. Analysing the positions of these mutations required a consistent co-ordinate system across all sample pairs. We therefore remapped the co-ordinates each variant discovered in an intergenic region to the co-ordinates of the ATCC 700669 reference genome. We used population-matched carriage isolates as the ancestral state to determine whether these mutations occurred in the blood or CSF isolate.

Fig. S10 shows all mutations plotted genome-wide in *S. pneumoniae*. The peaks correspond to mutations in genes described in Table 1. In the remaining 121 mutations in non-coding regions, we observed no clustering by position. Over all pairs of samples, intergenic mutations were spread between blood and CSF isolates when compared to a carriage reference isolate. This suggests none of the intergenic mutations were providing a selective advantage in either invasive niche.

The mutations in *N. meningitidis* are plotted in Fig. S11, 110 of which were in non-coding regions. We observed enrichment, but no niche specificity, in the upstream region of six genes. These mutations are listed in Table 3. Some of the mutations upstream of *porA* and *opc* were in phase-variable homopolymeric tracts, which are discussed more fully in the section below. The other mutations were upstream of the adhesins *hsf*/NMB0992 and NMB1994, which are involved in colonization [72] and immune interaction during invasion [73], and *frpB/*NMB1988, which is a surface antigen involved in iron uptake [74]. Differential expression of these genes may be an important factor affecting invasion, but the mutations we observed that may affect this do not appear to be specific to blood or CSF.

**Table 3.** Intergenic regions containing significantly repeated mutations between CSF and blood isolate pairs in *N. meningitidis* Ordered by increasing number of mutations; coordinates refer to the MC58 genome.

Coordinates	Downstream gene	Mutations between blood and CSF
1468329–1468331	*porA* (NMB1429)	7
1072215–1072328	*opc* (NMB1429)	7
1008872–1008985	*hsf* (NMB0992)	6
1315621–1315672	NMB1299	6
2092257–2092552	*frpB* (NMB1988)	5
2100124–2100258	NMB1994	4

### No evidence for repeated adaptation in phase-variable regions in *S. pneumoniae* and *N. meningitidis*

Phase-variable regions, which may also be intergenic, can mutate rapidly and are known to be a significant source of variation in pathogenic bacteria [75]. This mutation is an important mechanism of adaptation [76], and meningococcal genomes in particular contain many of these elements [77].

In *N. meningitidis*, we observed six samples with single base changes in length of the phase-variable homopolymeric tract in the *porA* gene’s promoter sequence, and five samples with the single base length changes in the analogous promoter sequence of *opc*. While changes in the length of these tracts will affect expression of the corresponding genes, both of which are major determinants of immune response [63, 78], the tract length does not correlate with blood or CSF specifically. Consistent with this, *porA* expression has previously been found to be independent of whether isolates were taken from CSF, blood or throat [63].

**Fig. 3.**
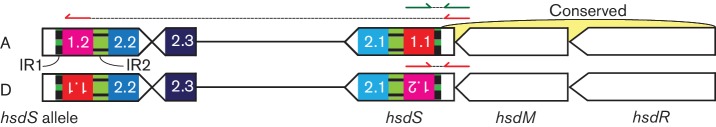
The structure of the *ivr* type I restriction-modification locus in *S.* *pneumoniae*. The restriction (*hsdR*) and methylation (*hsdM*) subunits, and the 5′ end of the specificity subunit (*hsdS*) are generally conserved. Inverted repeats IR1 (85 bp) and IR2 (333 bp) facilitate switching of downstream incomplete *hsdS* elements into the transcribed region. Top: the green read pair has the expected insert size, and suggests allele A (1.1, 2.1) is present; the red read pair is in the wrong orientation and has an anomalously large insert size. Bottom: the red read pair is consistent with the displayed inversion, suggesting allele D (1.2, 2.1) is present.

In *S. pneumoniae*, recent publications have highlighted a potential role in virulence for the *ivr* locus, a type I restriction-modification system with a phase-variable specificity gene allele of *hsdS* in the host specificity domain (Fig. 3) [18, 19, 79]. There are six possible different alleles A–F (Fig. S6) for *hsdS*, each corresponding to a different level of capsule expression. Some of these alleles are more successful in a murine model of invasion, whereas others are more successful in carriage. We used a mapping-based approach (see Methods) to determine whether any of these alleles were associated with either the blood or CSF niche specifically, which could be a sign of adaptation.

As the locus inversion is rapid and occurs within-host, we first ensured that cultured samples were representative of the original clinical samples using PCR quantification of each allele. However, as even a single colony contains heterogeneity at this locus, simply taking the allele with the most reads mapping to it in each sample gives a poor estimate of the overall presence of each allele in the blood and CSF niches. To take into account the mix of alleles present in each sample, and to calculate confidence intervals, we developed a hierarchical Bayesian model for the *ivr* allele (see Fig. S7 and Methods). This simultaneously estimated the proportion of each colony pick with alleles A–F for both each individual isolate (π), and summed over all the samples in each niche (μ). We applied this over *i* samples and *c* niches (in this case *c* can be blood or CSF).

**Fig. 4.**
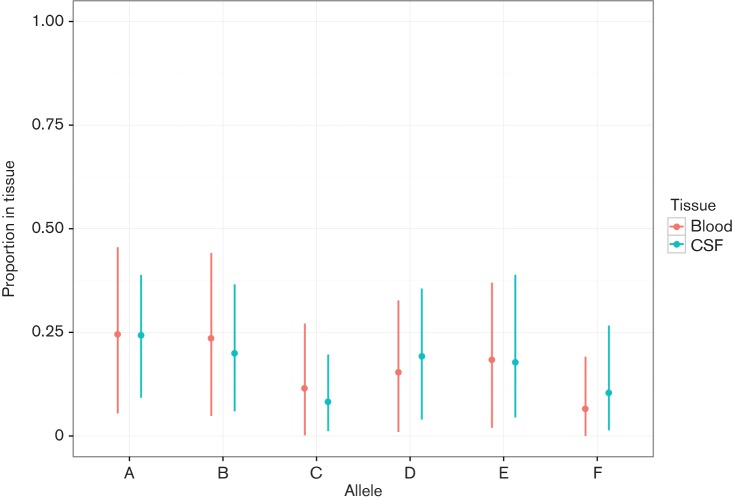
Mean and 95 % HPD for *μ_c_*. This shows the proportion of each allele present in each of blood and CSF tissues pooling across all samples.

For each pair of blood and CSF samples listed inTable S2, the difference in allele prevalence π_CSF_ − π_blood_ was calculated (Table S4). All *S. pneumoniae* samples had a difference in mean of at least one allele (as the confidence intervals overlap zero), highlighting the speed at which this locus inverts. While this means that between a single CSF and blood pair the allele at this locus usually changes, it is the mean of μ*_c_* (corresponding to the mean allele frequency in each niche over all sample pairs) that tells us whether selection of an allele occurs in either the blood or CSF more generally. This is plotted in Fig. 4. As the confidence intervals overlap, no particular allele is associated with either blood or CSF *S. pneumoniae* isolates.

Previous work on a murine invasion model [19] showed an increase in the proportion of alleles A and B over the course of infection. We did not observe the same effect in our clinical samples, though the large confidence intervals from the mathematical model suggest that genomic data with a small insert size relative to the size of repeats in the locus is limited in resolving changes in this allele. A small selective effect of the *ivr* allele between these niches would therefore not be detected, but we can rule out strong selection for a particular allele (odds ratio >2). Application to read data from a large carriage dataset may help resolve whether the same effect does occur in humans, as it would provide a greater temporal range over the course of pathogenesis.

## Discussion

Previous studies have shown that substantial levels of genomic DNA sequence variation occur in bacteria colonizing or infecting human hosts [12, 14, 15] and suggest that some of this variation may be due to selective adaptation [20, 22–24, 80]. Studies sampling from multiple sites over the course of an infection have been performed on other species of bacteria; while overall sequence similarity is generally found to be very high, some signs of adaptation to host niche have been found [23, 81, 82].

Such adaptations during invasive bacterial disease could lead to new insights into the processes of pathogenesis with the potential to inform therapies [83, 84]. Therefore, it is important to capture evidence of such variation; we sought to do this through large-scale genomic analysis. We have searched for variation in two different bacterial species comparing genomes from bacteria isolated from both blood and CSF from the same individuals in 869 bacterial meningitis cases.

We found overall that blood and CSF isolates have very similar genetic sequences. The small number of mutations observed are not randomly distributed throughout the genome, but are randomly distributed between blood and CSF isolates. These mutations are therefore an observation of a higher mutation rate in these regions during invasion, but not repeated adaptation to either the blood or CSF niche specifically. This result from a much larger sample size indicates that our previous observation of variation between blood and CSF isolates from a single case of meningitis was a rare event most likely driven by antibiotic selection pressure during treatment [24]. Supporting the conclusion from small genetic variations, no adaptation to either niche was observed in a focused analysis of structural variation in the *ivr* locus of *S. pneumoniae*. This is important as it captures some of the genome-wide epigenetic variation that affects virulence, via level of capsule expression [19]. Though not a sign of adaptation to the blood or CSF specifically, regions with a higher mutation rate may either be those under diversifying selection, such as the pilus in *N. meningitidis*, or provide a selective advantage outside of the nasopharynx.

The most notable of these observations for a locus not already known to be under diversifying selection is the *dlt* locus in *S. pneumoniae*. This locus controls levels on d-alanylation of cell wall teichoic acids. These are recognized by Toll-like receptors and have been shown to increase the sensitivity of the cytokine response of the host [64], while at the same time conferring resistance to host antimicrobial peptides (AMPs) [65]. In *Staphylococcus aureus*, mutation of the *dlt* locus has been shown to affect host tropism, though levels of d-alanylated teichoic acids did not correlate with infectivity [85].

We observed that after bloodstream invasion, the *dlt* operon accumulates significantly more damaging mutations than expected, implying a selection pressure against the gene function. This suggests that interaction with host AMPs changes upon shift from the nasopharynx to the invasive niche, a notion that is supported by *in vitro* observations that clinical isolates of *S. pneumoniae* are more susceptible to AMPs than carriage isolates [31]. We observed an excess of damaging mutations in *dlt* compared to other loci. That d-alanylation can be deleterious in invasive isolates has not been previously observed, though virulent isolates are known to be viable without this modification [86].

It is still possible that the genetics of the invading bacterium may already determine, prior to invasion, whether CSF invasion is possible, though previous studies at the gene level suggest this is not the case [29]. Future studies are needed to comprehensively identify whether adaptation occurs through genetic variation between carriage and invasion. This should be addressed through either comparing a large collection of pairs of carriage and invasive isolates with deep sequencing, or large-scale genome-wide association studies of carriage and disease collections. Alternatively, progression to meningitis could also be related to factors of the host, for example genetic factors affecting the immune system [87], or regulatory changes in the bacteria.

## Data Bibliography

1. The Wellcome Trust Sanger Institute. European Nucleotide Archive ERP004245 (2016).
2. Lees JA. GitHub https://github.com/johnlees/paired-samples (2016).
3. Lees JA. FigShare http://dx.doi.org/10.6084/m9.figshare.4329809 (2016).
4. Croucher NJ, Walker D, Romero P, Lennard N, Paterson GK *et al*. GenBank NC_011900.1 (2009).
5. Hoskins JA, Alborn W Jr, Arnold J, Blaszczak L, Burgett S *et al*. GenBank AE007317.1 (2001).
6. Denapaite D, Bruckner R, Nuhn M, Reichmann P, Henrich B *et al*. GenBank NC_013853.1 (2010).

## Supplementary Data

1264 Supplementary File 1
