# Supplementary material for: Large scale genomic analysis shows no evidence for pathogen adaptation between the blood and cerebrospinal fluid niches during bacterial meningitis
Source: Microb Genom. 2017 Jan 31;3(1):e000103. doi: 10.1099/mgen.0.000103 (PMC5361624; doi:10.1099/mgen.0.000103)
Supplement: Supplementary File 1 [file mgen-3-103-s001.pdf]

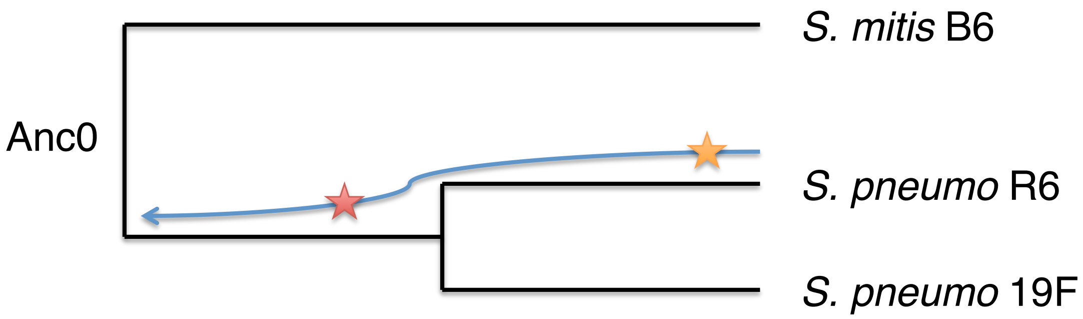

Figure S1: Tree topology from whole genome aligning two divergent *S. pneumoniae* genomes (R6 and 19F) and *S. mitis* B6. The rates of SNP and INDEL accumulation were taken by averaging all events along the branch Anc0->R6. A small number of mutations (mean 200) were simulated using the rates along this branch.

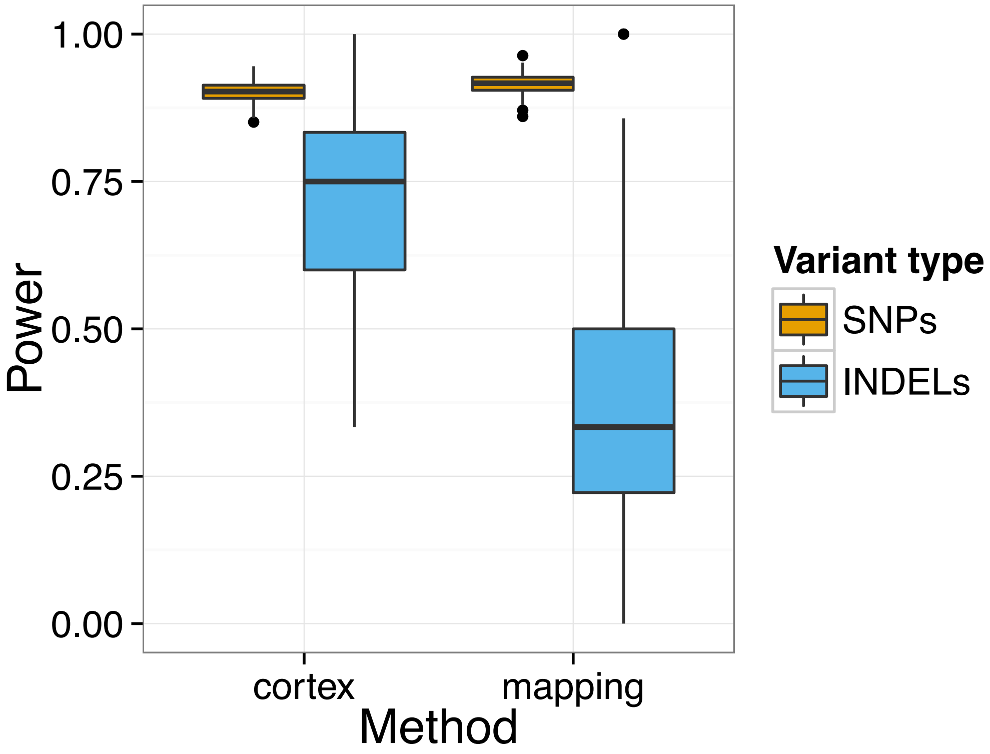

Fig S2: Boxplot of power (recall) for each method of variant calling for 100 simulated samples. Cortex and mapping to *de novo* assemblies/hybrid are shown separately for SNPs (gold) and INDELs (blue). False positive rate is shown in Figure S6.

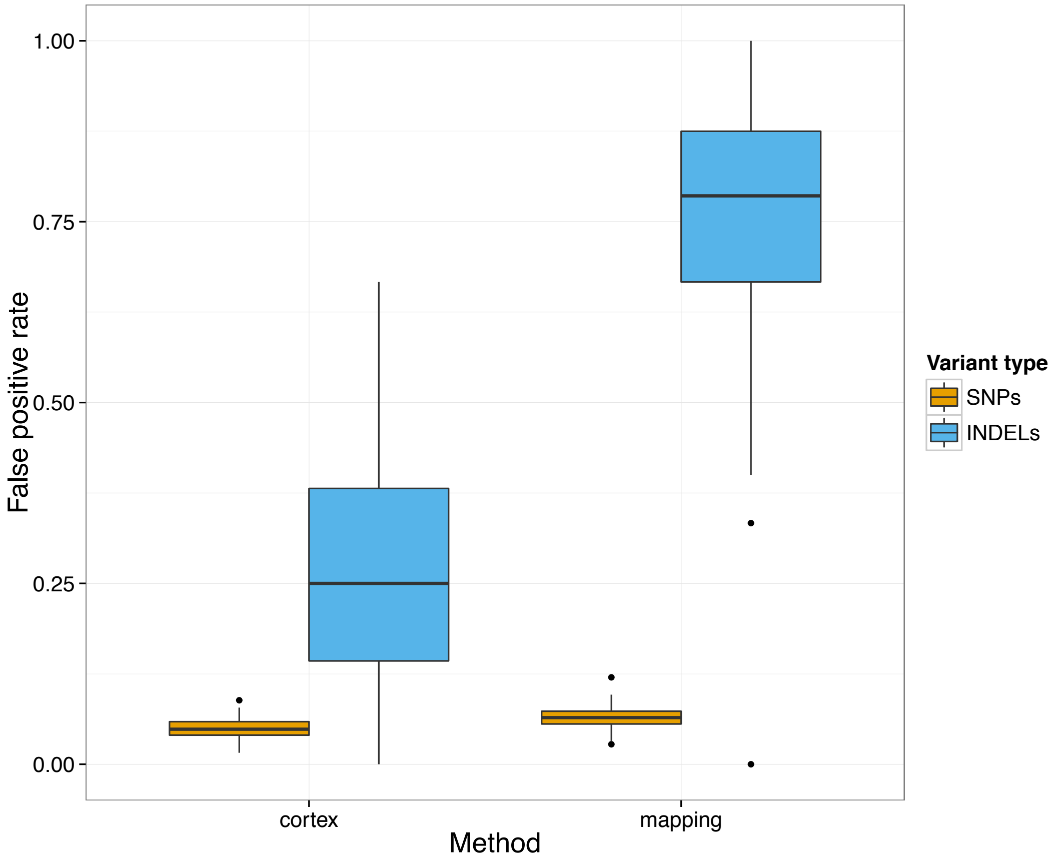

Figure S3: Box plot of false positive rate (or false discovery rate, FDR) for each calling method. Run on the same 100 simulated samples as Figure 1, calculated by number of false positives/number of true positives. FDR for SNPs (yellow) and INDELs (blue) are shown separately.

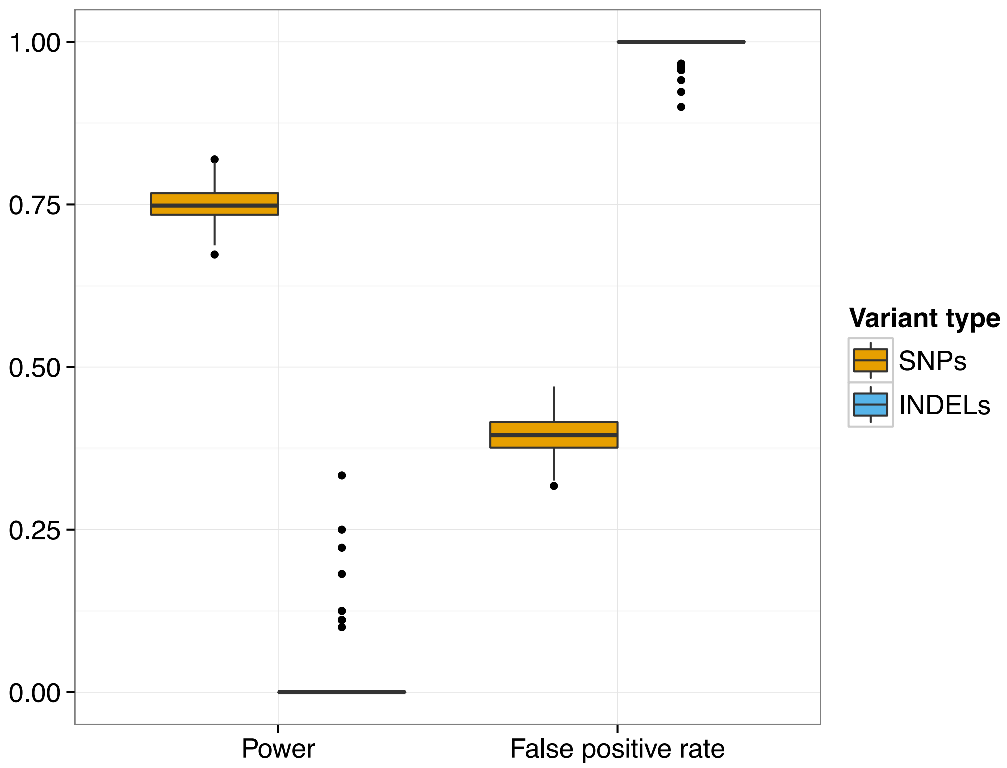

Figure S4: Box plot of power and false positive rate for reference based calling. Run on the same 100 simulated samples as Figure 1, calculated by number of false positives/number of true positives. SNPs (yellow) and INDELs (blue) are shown separately.

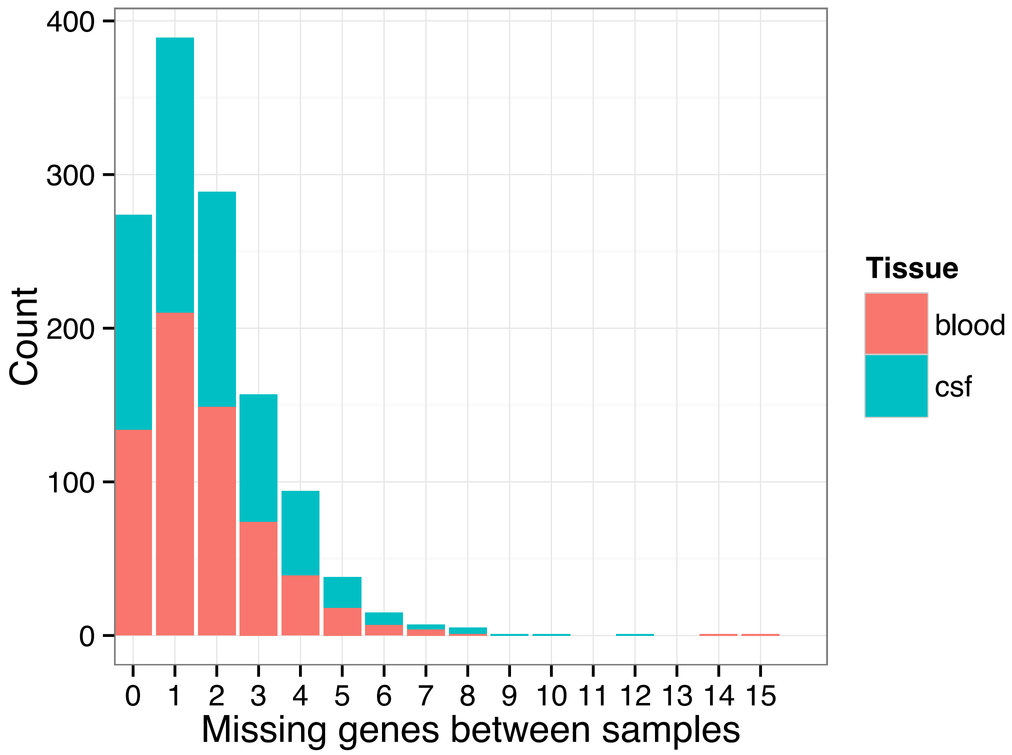

Figure S5: Count of annotated genes present in blood but not CSF (red) or vice-versa (turquoise) between the 674 *S. pneumoniae* samples. The level of variation is inflated compared to Figure 3 due to frequent misannotation of CDSs.

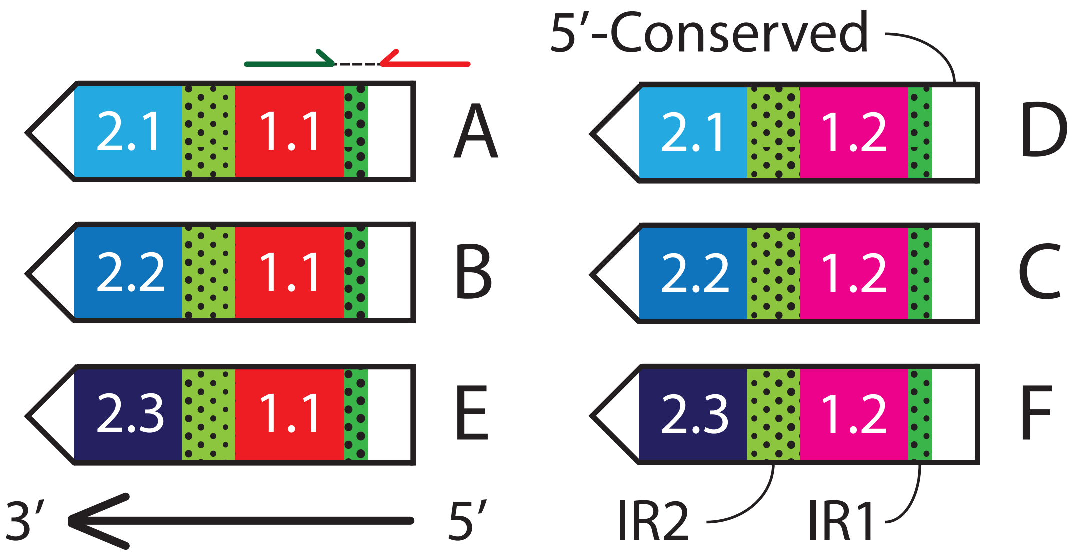

Figure S6: Possible alleles A-F for the *hsdS* gene, and the segments 1.i and 2.j they are made up from. Also shown: inverting repeats mediating phase variation, read pairs mapping from conserved regions to variable alleles.

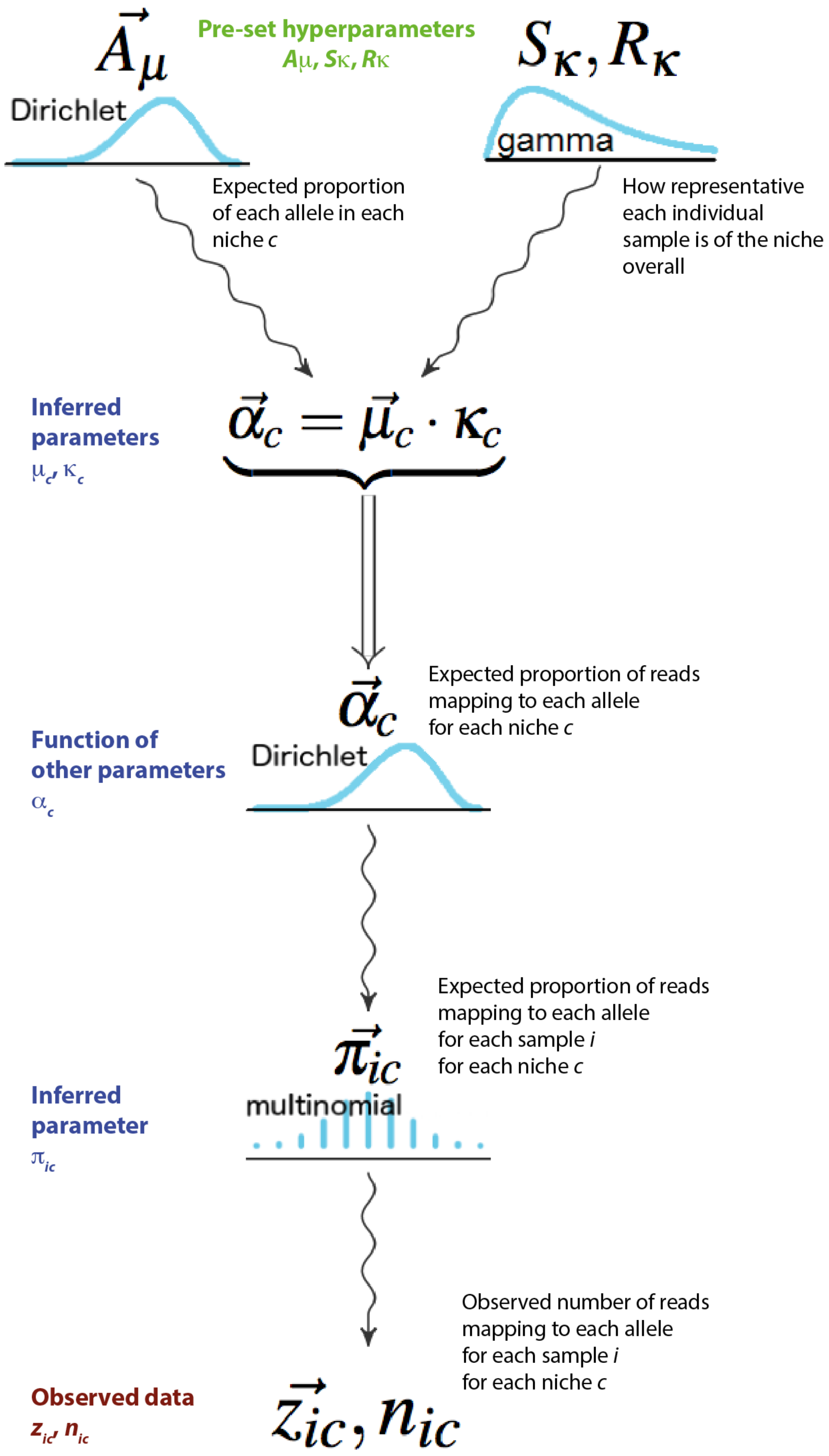
Pre-set hyperparameters
Aμ, Sκ, Rκ
Dirichlet
gamma
Expected proportion of each allele in each niche c
How representative each individual sample is of the niche overall
Inferred parameters
μc, κc
Function of other parameters
αc
Dirichlet
Expected proportion of reads mapping to each allele for each niche c
Expected proportion of reads mapping to each allele for each sample i for each niche c
Inferred parameter
πic
multinomial
Observed number of reads mapping to each allele for each sample i for each niche c
Observed data
zic, nic

29

Fig. S7: Hierarchical model for *hsd* allele. Solid double arrows denote a deterministic relationship; wavy arrows represent a value drawn from a distribution. *z* is a vector of the number of reads mapping to each allele from a total of N reads mapping to the variable region; *i* is the sample number; *c* is an index for tissue type. μ_c, κ are hyperparameters for mean allele prevalence and how closely a sample is representative of a tissue type respectively. $A\mu$, $B\mu$ are priors for allele prevalence in invasive disease. $S\kappa$, $R\kappa$ are the shape and rate parameters for a gamma distribution, which were used to set a broad prior on κ.

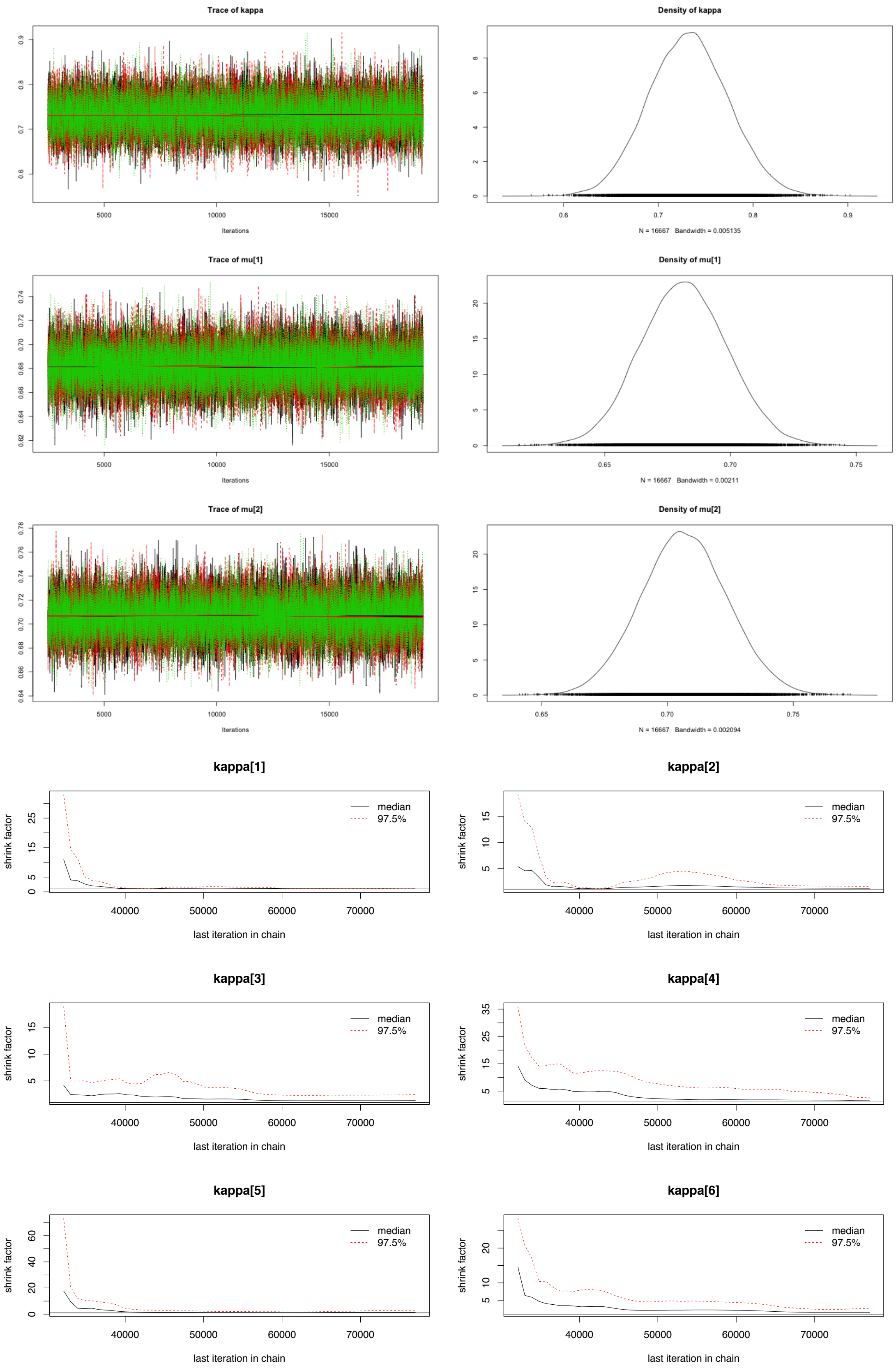

Figure S8: Diagnostics of MCMC from R-M allele model. Above left panels: Plot of chain position and mean of κ and μ for the model of allele 1.i, for the three chains

run. Right panels: Samples and posterior density for these three parameters. Bottom: Shrink factor (Gelman-Rubin convergence diagnostic) at each sampling iteration for $\kappa_{A\text{-}F}$ over three chains. A value of 1 suggests the chain has converged.

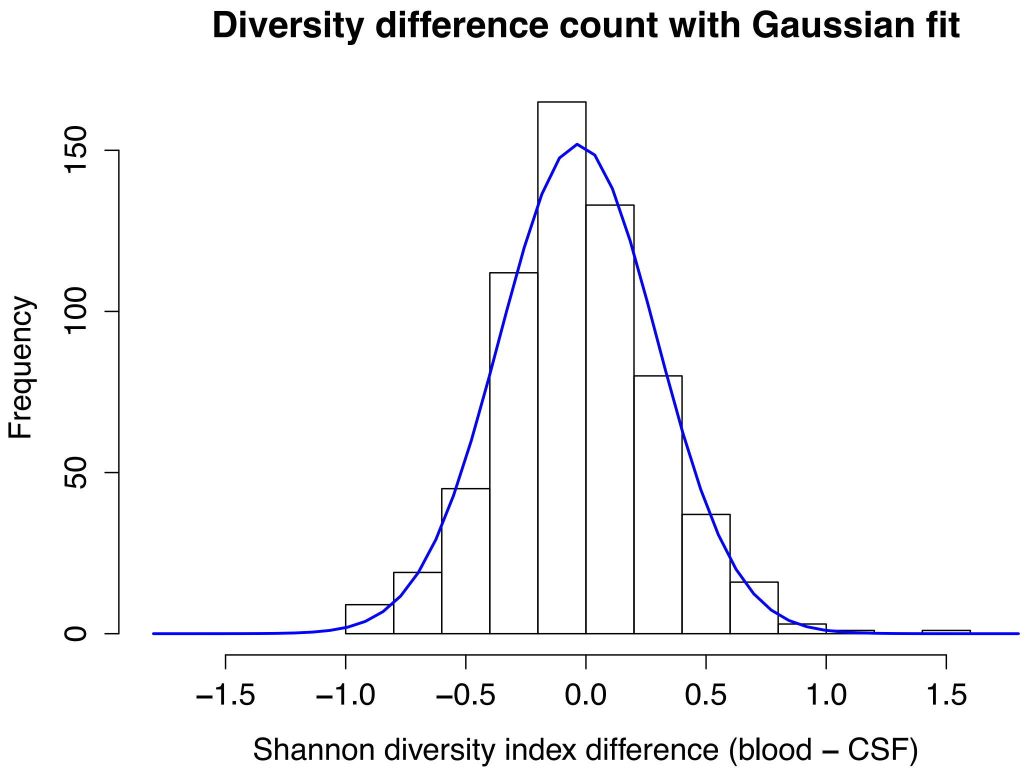

Figure S9: Distribution of difference in Shannon diversity index between the *ivr* locus model π_{blood} and π_{CSF}. A Gaussian distribution is fitted to the data, which has a mean of roughly zero and little skew. The maximum possible Shannon diversity index (for equal amounts of each allele A-F) is 1.8.

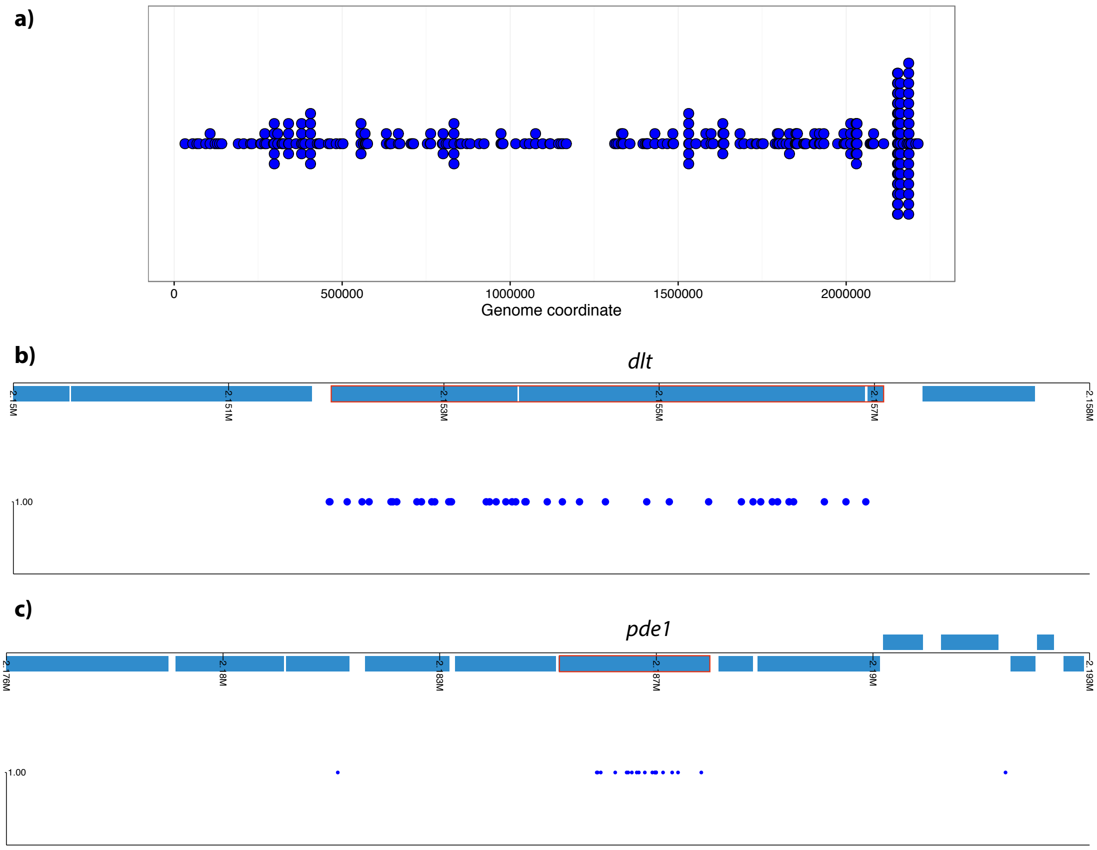

Figure S10: Mutations observed between all *S. pneumoniae* pairs, overlaid onto the Spn23F reference. Each blue point on the lower row corresponds to a SNP or INDEL variant observed between at least one sample pair. The blocks in the upper row represent CDSs, lying above or below the central line depending on whether they are on the forward or reverse strand respectively. The facets show **a)** whole genome (stacked, grouped by 1000 bp windows); **b)** *dlt* operon (four genes in the centre, from 2 152 238 to 2 156 543 base pairs); **c)** *pde1* (gene in the centre from 2 185 398 to 2 187 371 base pairs).

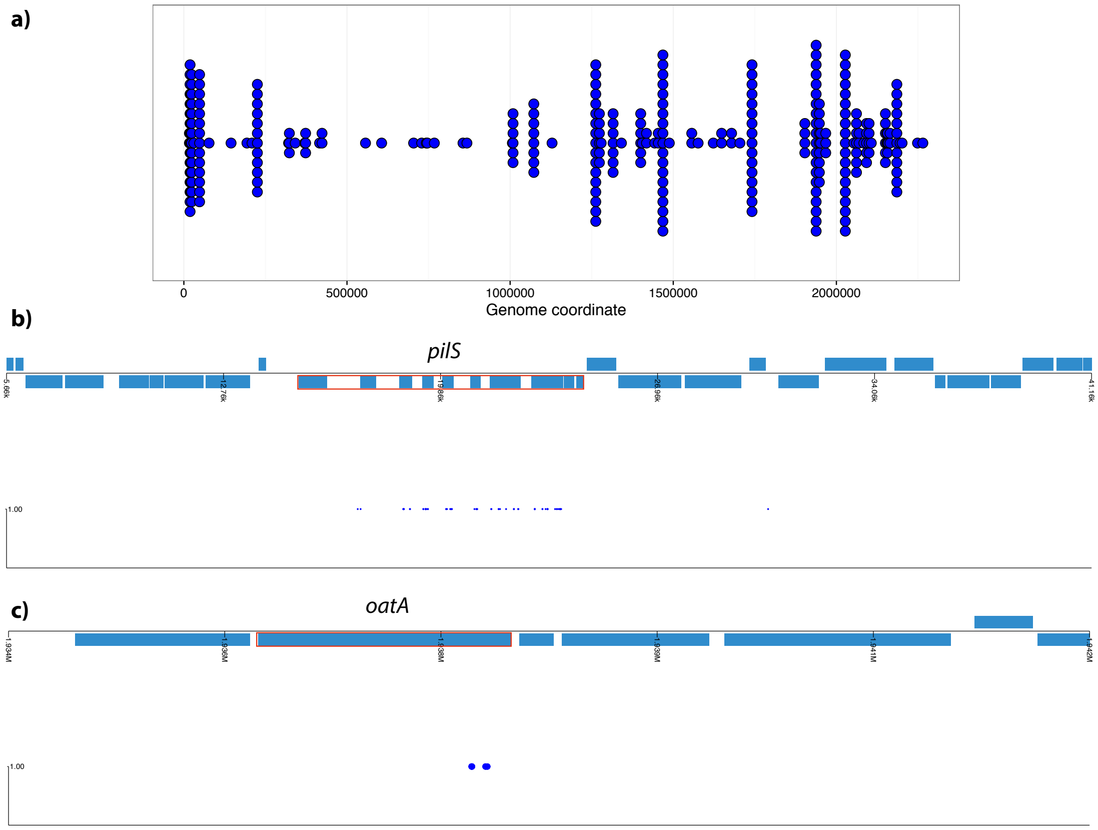

Figure S11: Mutations observed between all *N. meningitidis* pairs, overlaid onto the MC58 reference. As figure S3. **a)** whole genome; **b)** pilus encoding genes. Mapping to the MC58 reference places these incorrectly in the unexpressed *pilS* cassette (Figure S5b); compared to the reference the isolates have recombined between *pilS* and the expressed *pilE*.

Table S1: Number of each bacterial species sequenced in the study, and the average sequencing coverage.

Organism	Number of blood/CSF pairs sequenced	Number of nasopharynx/CSF pairs sequenced	Mean sequencing coverage
Streptococcus pneumoniae	674	6	91.7
Neisseria meningitidis	195	48	96.6

Table S2: Samples containing a full *ivr* locus. 621 sample pairs where both the blood and CSF isolates have at least one read mapping to both the 5' and 3' end of the *ivr* locus.

2000011
2000017
2000018
2000019
2000037
2000109
2000134
2000139
2000157
2000162
2000164
2000165
2000180
2000188
2000223
2000254
2000267
2000271
2000290
2000305
2000335
2000353
2000390
2000391
2000404
2000405
2000475
2000501
2000596
2000722
2000727
2000770
2000810
2000817
2000830
2000854

2000900
2000977
2001006
2001012
2001014
2001047
2001096
2001128
2001141
2001151
2001157
2001192
2001338
2001398
2001507
2001517
2001580
2001589
2001634
2001755
2001762
2001771
2001815
2001843
2001854
2001864
2001865
2001866
2001922
2001984
2002008
2002054
2002096
2002158
2002167
2010007
2010019
2010032
2010053
2010066
2010096
2010105

2010135
2010184
2010256
2010271
2010282
2010337
2010388
2010433
2010453
2010459
2010460
2010470
2010523
2010572
2010650
2010658
2010667
2010710
2010795
2010813
2010827
2010835
2010838
2010917
2010983
2011049
2011124
2011340
2011440
2011733
2011795
2012019
2012064
2012157
2012273
2012283
2012311
2012361
2012559
2012586
2012612
2020051

2020055
2020111
2020159
2020169
2020172
2020316
2020375
2020452
2020457
2020555
2020756
2060375
2060406
2060519
2060558
2060698
2060702
2060776
2060788
2060793
2060837
2060849
2060880
2060915
2060921
2060949
2060981
2060997
2061015
2061049
2061207
2061274
2061287
2061343
2061365
2061381
2061425
2061438
2061532
2061564
2061586
2061627

2061651
2061666
2061708
2061718
2070004
2070010
2070039
2070043
2070059
2070111
2070122
2070154
2070203
2070227
2070237
2070241
2070243
2070268
2070302
2070309
2070331
2070340
2070366
2070370
2070375
2070380
2070456
2070497
2070508
2070532
2070563
2070614
2070661
2070673
2070717
2070746
2070761
2070779
2070782
2070822
2070853
2070900

2070942
2070980
2070986
2071003
2071012
2071052
2071059
2071078
2071102
2071119
2071142
2071207
2071265
2071266
2071298
2071338
2071358
2071408
2071460
2071493
2071544
2071750
2071802
2071810
2071874
2071889
2071920
2071939
2072050
2080022
2080023
2080027
2080054
2080058
2080061
2080063
2080078
2080107
2080142
2080165
2080169
2080170

2080186
2080190
2080239
2080304
2080323
2080353
2080426
2080442
2080524
2080526
2080565
2080611
2080628
2080652
2080660
2080671
2080725
2080748
2080749
2080767
2080780
2080828
2080859
2080977
2081033
2081046
2081077
2081082
2081097
2081103
2081106
2081229
2081302
2081417
2081421
2081618
2081627
2081738
2081959
2081987
2082075
2082125

2082126
2082140
2082170
2082171
2082187
2082239
2082278
2090002
2090008
2090009
2090026
2090043
2090069
2090098
2090103
2090243
2090250
2090270
2090274
2090297
2090323
2090334
2090355
2090521
2090544
2090546
2090568
2090575
2090582
2090593
2090841
2090885
2090905
2090930
2090932
2091127
2091161
2091165
2091204
2091249
2091263
2091291

2091623
2091680
2091687
2091748
2091775
2091794
2092630
2093028
2093029
2093039
2093049
2093090
2093367
2093387
2093449
2093895
2094015
2094025
2094034
2094035
2094064
2094079
2094095
2094096
2094131
2094185
2094205
2094232
2094236
2094414
2094468
2094469
2094473
2094482
2100006
2100011
2100023
2100047
2100115
2100118
2100170
2100183

2100187
2100194
2100208
2100235
2100257
2100261
2100262
2100281
2100284
2100299
2100302
2100317
2100464
2100484
2100506
2100507
2100533
2100555
2102095
2102096
2102097
2102233
2102254
2102256
2102257
2102535
2102551
2102588
2102608
2102612
2102616
2102632
2102700
2102708
2102719
2102722
2102736
2102737
2102993
2103136
2103275
2103279

2103386
2103415
2103421
2103449
2103457
2103495
2103547
2103561
2103573
2103590
2103602
2103629
2103634
2103678
2103734
2103738
2103765
2104279
2104318
2104330
2104338
2104340
2104348
2104370
2110014
2110087
2110097
2110168
2110185
2110192
2110252
2110270
2110371
2110374
2110390
2110393
2110411
2110469
2110476
2110499
2110517
2110530

2110548
2110559
2110563
2110613
2110618
2110650
2110663
2110701
2110740
2110785
2110786
2110803
2110816
2110873
2110892
2110898
2110956
2111062
2111071
2111108
2111114
2111115
2111189
2111203
2111269
2111274
2111278
2111297
2111306
2111309
2111376
2111417
2111460
2111474
2111505
2111558
2111578
2111604
2111609
2111619
2120014
2120019

2120053
2120068
2120087
2120095
2120097
2120109
2120142
2120175
2120206
2120296
2120303
2120310
2120326
2120346
2120363
2120400
2120452
2120526
2120594
2120603
2120681
2120725
2120824
2120836
2120846
2120891
2120954
2120994
2121022
2121064
2121275
2121393
2121456
810516
821147
821308
840526
860466
861280
861345
861716
870191

870249
871016
880391
880683
882037
892663
900991
920822
930845
941051
960548
962160
970229
980161
981297
981749
981776
981819
981830
981842
982024
982055
982060
982064
982080
982134
982142
982238
982320
982346
990045
990066
990083
990109
990110
990285
990341
990357
990389
990444
990460
990473

990506
990589
990621
990631
990696
990728
990812
990828
990937
990957
991066
991067
991071
991098
991102
991103
991145
991155
991170
991204
991233
991393
991401
991525
991687
991707
991756
991757
991800
991861
991881
991882
991905
991921
991925
992005
992020
992078
992168

70

Table S3: Predicted effect of variants in the *dlt* operon with respect to the R6 reference (36/38 called mapped), and the sample (blood or CSF) the variant is found in. Predicted effect is as defined in the Sequence Ontology (http://www.sequenceontology.org/). Important examples include 'stop_gained' where a codon is changed resulting in a premature stop and shortened transcript, and 'stop_lost' where a base in the stop codon is changed resulting in an elongated transcript. The variant in 981297 is a single large deletion affecting both *dltB* and *dltC*.

Sample	Gene	Predicted effect	Sample with variant
2000109	*dltB*	frameshift_variant	csf
2000134	*dltD*	frameshift_variant	csf
2000770	*dltD*	missense_variant	csf
2001866	*dltD*	stop_gained	blood
2010032	*dltB*	frameshift_variant	blood
2020159	*dltA*	missense_variant	csf
2020756	*dltD*	missense_variant	blood
2060558	*dltD*	stop_gained	csf
2060981	*dltA*	missense_variant	blood
2060981	*dltB*	frameshift_variant	blood
2061666	*dltC*	frameshift_variant	blood
2070194	*dltA*	frameshift_variant	blood
2070243	*dltA*	missense_variant	csf
2070331	*dltB*	missense_variant	csf
2080611	*dltB*	stop_gained,inframe_insertion	csf
2080977	*dltD*	synonymous_variant	csf
2080977	*dltA*	missense_variant	csf
2090521	*dltA*	frameshift_variant	blood
2094232	*dltA*	missense_variant	blood
2100161	*dltC*	stop_gained	blood
2100161	*dltD*	missense_variant	blood
2110087	*dltA*	stop_lost,inframe_deletion	blood
2110650	*dltB*	stop_gained	csf

2111558	*dltB*	frameshift_variant,stop_lost	blood
2120994	*dltA*	missense_variant	blood
860466	*dltC*	frameshift_variant	csf
870249	*dltB*	frameshift_variant	blood
971916	*dltD*	missense_variant	blood
971916	*dltB*	missense_variant	csf
981297	*dltC*	coding_sequence_variant,5_prime_UTR_variant	blood
981297	*dltB*	coding_sequence_variant,3_prime_UTR_variant	blood
982134	*dltA*	stop_gained	blood
990444	*dltD*	stop_gained	csf
990460	*dltB*	frameshift_variant	blood
991525	*dltD*	frameshift_variant	blood
991756	*dltD*	synonymous_variant	blood

Table S4: Number of pairs which have 95% HPD intervals of π_{CSF}-π_{Blood} not overlapping zero. Suggests that a different amount of the allele is present in each isolate.

Allele	Discordant pairs	Percentage of total
Any	621	100%
A	481	77.46%
B	493	79.39%
C	532	85.67%
D	511	82.29%
E	465	74.88%
F	539	86.80%
